# Investigation of antioxidants and intrinsic factors as the cause of discoloration in vacuum-packaged beef

**DOI:** 10.1007/s44463-026-00099-0

**Published:** 2026-08-21

**Authors:** Johannes Krell, Theresa Müller, Cora Schmetzer, Alejandro Poveda-Arteaga, Jochen Weiss, Nino Terjung, Monika Gibis

**Affiliations:** 1 Department of Food Material Science, University of Hohenheim, Stuttgart, Germany; 2 German Institute of Food Technology, Quakenbrück, Germany

**Keywords:** Beef, Myoglobin, Discoloration, Packaging, Vacuum

## Abstract

Intrinsic factors of vacuum-packaged, discolored beef samples found in the early stages of wet aging at the slaughterhouse were measured and compared to a control group. The aim was to identify possible differences in intrinsic factors that could explain the discolorations. L*, a*, b* values, and myoglobin redox state levels were measured to characterize the extent of discoloration and the oxidation state of myoglobin. pH value, redox potential, total reducing activity, and the concentrations of lactate, *α*-tocopherol, *β*-carotene, and NAD^+^/NADH were analyzed to identify differences. Significant (*p* < 0.05) differences and correlations were observed in color values and myoglobin redox states between the control and discolored samples, e.g., a* = 11.21 (control) and 9.42 (discolored); metmyoglobin = 0.79 (control) and 1.01 (discolored). Except for directly color-related parameters, only a significant difference in lactate concentration was observed (3.90 mg/g (control) and 5.60 mg/g (discolored) without a significant change in pH [5.60 (control) and 5.61 (discolored)]. Since lactate is reported to stabilize the color of meat rather than causing discoloration, the cause of the discoloration might be attributed to a multifactorial interaction of intrinsic parameters, or extrinsic parameters during the slaughtering, deboning, cutting, and packaging process. Although the cause of the discoloration could not be identified, this study displays important data on the interactions between intrinsic parameters. Therefore, this study can be considered an exploratory investigation that lays the foundation for further research to identify the causes of discoloration.

## Introduction

The so far unexplained, irreversible discoloration of vacuum-packaged beef during the first few days of the wet-aging process can be observed in multiple German slaughterhouses. These incidents are irregular and unpredictable. However, they occur frequently enough to cause financial damage, as consumers reject discolored meat (Thies et al. 2024, 2025; Poveda et al. 2025; Gibis and Terjung 2021). From ecological, economic, and animal welfare perspectives, it would be beneficial to identify the cause of the discoloration and, ideally, develop prevention methods.

The current literature on the irreversible discoloration of vacuum-packaged beef during the early stages of wet aging is very limited. In a similar study, Poveda et al. (2025) investigated the discolorations in the same slaughterhouse under the same conditions, focusing on the microbiome. However, a clear connection between the microbiome and the discoloration could not be confirmed. Thus, the aim of this study was to investigate intrinsic muscle parameters which are positively or negatively connected to meat discoloration and determine whether deviations in these parameters cause discoloration.

L*a*b* color value and myoglobin (Mb) level determination are useful methods for characterizing the color and redox state of myoglobin, and thus the extent of discoloration. The L*a*b* color values describe the color of the samples in the CIELab color space. The L* value describes the lightness (0–100), the a* value the green-red axis (− 50 to 50), and the b* value the blue-yellow axis (− 50 to 50) (Wieser 2010; King 2023). The three important redox states of Mb are the base form, deoxymyoglobin (DMb) (no ligand, central iron atom Fe^2+^), the oxygenated form oxymyoglobin (OMb) (O_2_ bound, central iron atom Fe^2+^), and the oxidized form metmyoglobin (MMb) (H_2_O bound, central iron atom Fe^3+^) (King 2023; Krell et al. 2024, 2025, 2026).

The pH value and lactate concentration provide insight into postmortem glycogen metabolism deviations, which can indicate the stress levels of cattle before slaughter (Coombes et al. 2014; Devine and Dikeman 2014; Matarneh et al. 2023). These parameters can also be affected by lactic acid bacteria, the dominant microbes in vacuum-packaged beef (Ercolini et al. 2011; Lavieri and Williams 2014; Pennacchia et al. 2011). High pH deviations are associated with meat discoloration (Coombes et al. 2014; Devine and Dikeman 2014; Matarneh et al. 2023). While excessive lactic acid bacteria are definitely connected to meat spoilage, connections to meat discoloration have not been confirmed in the literature (Ercolini et al. 2011; Pennacchia et al. 2011; Hanna et al. 1983).

The redox potential can be used as an indication of the general oxidation state of the muscles. Higher values indicate an increased oxidation potential of the tissue being measured (Cucci et al. 2020).

The discoloration of meat is caused by Mb oxidation to MMb (King 2023; Mancini 2013). When it comes to oxidative processes in meat, more than just Mb needs to be considered. Other proteins and lipids can also be oxidized (Clausen et al. 2009; Meng et al. 2026). During lipid oxidation radicals are formed, that can further promote Mb oxidation in a chain reaction (Meng et al. 2026). There are multiple processes that can prevent or reverse the oxidation in the muscles, which retains Mb functionality, keeps the oxygen transport chain to the mitochondria intact, and allows continuous oxidative phosphorylation, thus keeping the cell alive (Weibel 1984; Bekhit and Faustman 2005; Mitacek et al. 2019; Nassu et al. 2011).

There are different measures that describe a muscles ability to reverse oxidative processes. Total reducing activity (TRA) of tissue describes its overall non-specific reduction potential. TRA includes all enzymatic (including mitochondria-mediated) or non-enzymatic components in the tissue that exhibit reducing properties toward metmyoglobin and other components such as lipids and other proteins (Tuell et al. 2021). TRA is a dimensionless parameter, and higher values indicate greater reducing activity (Tuell et al. 2021; Lee et al. 1981). Another way to describe the reducing ability of meat is the metmyoglobin reducing ability (MRA). Like TRA, MRA includes all enzymatic (including mitochondria-mediated) or non-enzymatic components in the tissue with reducing properties. However, the reducing properties are limited to metmyoglobin reduction (Mitacek et al. 2019). Since oxidative processes in meat include not only to myoglobin oxidation, but also lipid and other protein oxidation, and since oxidation products can lead to further oxidation, TRA was used in this study, to determine the reducing potential of the samples (Clausen et al. 2009; Mitacek et al. 2019).

The muscle tissue’s mechanism for preventing oxidation works via antioxidant radical quenchers, such as *α*-tocopherol and *β*-carotene (Nassu et al. 2011; Jin et al. 2015; Krell et al. 2026). While *β*-carotene is more prevalent in fat tissue and organs, *α*-tocopherol plays a crucial role in preventing oxidation because it is incorporated into muscle cell membranes, thereby quenching radicals that could enter the cell (Nassu et al. 2011; Jin et al. 2015). If the discoloration was connected to low antioxidant content, it would be a favorable since past studies have shown that the antioxidant content in muscle tissue can be increased by antioxidant supplementation to the feed (Nassu et al. 2011; Faustman et al. 1989).

If myoglobin has already been oxidized, then mitochondrial enzyme systems, such as NADH-cytochrome b5 reductase, can reduce it back to DMb (Bekhit and Faustman 2005; Elroy et al. 2015). These enzyme systems require the reduction equivalent, nicotinamide adenine dinucleotide hydride (NADH) to reduce MMb to DMb (Bekhit and Faustman 2005; Elroy et al. 2015). After the reduction process, the NADH turns into its oxidized state NAD^+^ (Weibel 1984). After slaughter, NADH cannot be regenerated due to a lack of ATP production. Therefore, quantifying NADH/NAD^+^ is a useful method for assessing overall enzymatic reduction capacity of the muscle tissue, as well as the amount of already oxidized reduction equivalents (Weibel 1984; Bekhit and Faustman 2005; Mitacek et al. 2019). Low overall reduction capacity or high NAD^+^ and low NADH concentrations could indicate low color stability (Bekhit and Faustman 2005; Mitacek et al. 2019).

The influence of mitochondria on meat color can be ambiguous. As already described, there are mitochondria-mediated reducing processes that, under normal metabolic conditions, can reverse Mb oxidation and therefore stabilize meat color (Mitacek et al. 2019). These systems also reduce reactive oxygen species (ROS) formed during mitochondrial energy metabolism, where oxygen is used to generate ATP from pyruvate, malate, or succinate (Ke et al. 2017). According to Ke et al. (2017), the lack of blood flow after slaughter leads to decreased mitochondrial reducing efficiency. This results in an accumulation of ROS, which can trigger lipid oxidation. Depending on the state of the mitochondrial reducing systems, a high oxygen consumption rate may result in rapid ROS accumulation (Mitacek et al. 2019; Ke et al. 2017).

All of the mentioned processes are influenced by the packaging conditions of the meat. If the storage temperature is relatively high, the rate of oxidative reactions increases (Mancini 2024; Rogers et al. 2014). Insufficient vacuum application to the packages not only allows general oxidation, but also increases oxygen consumption rates in the mitochondria. This can lead to increased overall oxidation due to ROS formation (Tuell et al. 2021; Krell et al. 2026).

The hypothesis of this study is that one or more of the intrinsic factors in the discolored muscle tissue differ from those in control muscle tissue, and that one or more of these factors caused the discoloration.

## Materials and methods

### Sample collection and preparation

Discolored beef samples from the hind that are typically removed and vacuum-packaged in one piece for wet-aging and further distribution, consisting of *M. gluteobiceps*,* M. tensor fasciae latae*,* and M. vastus lateralis* were obtained from a German slaughterhouse (Co. Böseler Goldschmaus, Garrel, Germany). The samples were collected over a period of four months on thirty different days by screening for discolored samples in the wet-aging facility of the slaughterhouse. The discolored samples were collected vacuum-packaged from the wet-aging aging facility of the slaughterhouse 5–8 days postmortem. If discolored samples were found, control samples for reference were also removed from the same package. The muscles in each package are guaranteed to come from the same livestock breeder and to have been raised and slaughtered under the same conditions. The discolorations that were investigated could not be replicated a laboratory setting. For this reason, the sampling had to be conducted in a commercial slaughterhouse. The discolorations occurred rarely and with a delay after packaging. Therefore, attributing the muscle pieces to their respective animals in the 25 kg vacuum bags after 1–2 days of storage, was not possible. The authors could interfere with the ongoing slaughtering operation only to a limited extent and for a limited time. This explains the sample size and the use of an independent sampling approach instead of a paired one. After identification, the samples were vacuum-packed, frozen at – 18 °C, and transported to the research facility for further analysis. During the study, nine discolored samples and nine reference samples from 18 different animals were obtained. A detailed description of sample preparation and collection at the slaughterhouse is described by Poveda et al. (2025).

### Sample measurement

*Photographs of the samples:* The frozen samples were thawed for two days at 2 °C. Then, the packaged samples were photographed using an EOS 250D camera (Co. Canon, Japan) and a camera holder described by Ruedt et al. (2020).

*Reflectance and color measurement: *The reflectance spectra (for the relative myoglobin redox state calculation) and the color of different points at the surface of the samples were measured in quintuplicate using a spectrophotometer (UltraScan VIS 1091, illuminant xenon, diffuse/8° observer angle, Co. HunterLab, Reston, VA, USA).

*pH measurement:* The samples were removed from the vacuum packaging, and the pH value was measured in triplicate using a pH probe for meat (WTW pH 537, Co. Xylem Analytics, Weilheim, Germany).

*Analysis of antioxidants:* Control and discolored muscle tissue were then removed for further chemical analysis. The *α*-tocopherol and *β*-carotene concentrations were measured in quadruplicate via ultra-high-performance liquid chromatography (UHPLC) (Thermo Scientific Vanquish UHPLC, Co. Thermo Fisher, Waltham, MA, USA) according to the method of Stuetz and colleagues (Stuetz 2016). The method of Grebenstein and Frank (2012) was used for the extraction of the analyzed compounds. The extraction method was slightly adjusted. The samples were homogenized using a SilentCrusher (Co. Heidolph, Schwabach, Germany) for 1 min at 9000 rpm. The homogenized samples were saponified with saturated KOH at 70 °C in a shaking water bath for 30 min. Afterwards the samples were neutralized using glacial acetic acid. Then n-hexane was added and the samples were mixed for 1 min by hand inversion. Afterwards the samples were centrifuged at 1000 rpm for 3 min to allow phase separation and the supernatant was separated. The n-hexane extraction step followed by phase separation was repeated three times in total. The combined supernatants were dried under nitrogen with a nitrogen evaporator (Vapotherm Mobil III Evaporator, Co. Barkey, Griesheim, Germany), the residues were resuspended in 300 µL of ethanol. Except for the resuspension of the residues in ethanol all remaining steps including the given volumes were performed as described in the method of Grebenstein and Frank (2012). To protect the vitamins from photo oxidation the blinds in the lab were closed, the lights turned off, and a fume hood covered in dark plastic foil was used as a working area.

*Measurement of total reducing ability:* The total reducing ability of the samples was analyzed in duplicate according to the method described by Lee et al. (1981) without deviations using a SilentCrusher (Co. Heidolph, Schwabach, Germany) for homogenization and the UV5 photometer (Co. Mettler-Toledo, Murnau, Germany) for measurement.

*Measurement of redox potential:* For the determination of the redox potential, 5 g of each sample was weighed in triplicate in centrifuge tubes. Then, 15 mL of a 0.01 M sodium phosphate buffer (pH 6.3) was added. The samples were homogenized using a SilentCrusher (Co. Heidolph, Schwabach, Germany) for 1 min at 9000 rpm, followed by centrifugation for 15 min at 4 °C and 2000 rcf (×g). The supernatant was first filtered using a folded filter and then filtered by using a CHROMAFIL RC syringe filter, pore size 0.45 μm (Co. Macherey-Nagel, Düren, Germany). Lastly, 10 mL of 0.01 M sodium phosphate buffer was added to increase the total volume for easier measurement. The redox potential of each sample was measured using a Redox/ORP Sensor (Co. SI Analytics/Xylem Analytics, Weilheim, Germany) for 2 min until equilibrium was reached. After the measurement, the redox electrode was placed in a calibration solution until it reached 470 mV to guarantee the same starting point for all measurements.

*Analysis of NAD*^+^*/NADH:* The NAD^+^/NADH concentrations were analyzed in quadruplicate using the NAD^+^/NADH Assay Kit (Prod. Nr. MAK468, Co. Sigma-Aldrich). The homogenization of the muscle tissue was performed in phosphate buffered saline from the assay kit, using a Digital Disruptor Genie (Co. Scientific Industries, New York, USA) and Roti SampleLyse Soft Tissue 1 tubes (Co. Carl Roth, Karlsruhe, Germany) for 10 min at 2700 rpm.

*Analysis of lactate: *The lactate concentrations were measured in quadruplicate via high-performance liquid chromatography (HPLC) (Co. Agilent 1100 series, Agilent Technologies, Santa Clara, USA) according to the extraction and measurement methods of Walz and colleagues (Walz et al. 2017). The injection volume was 20 µL at a flow rate of 0.7 ml/min and a temperature of 35 °C. The column used was a Reprosil pur, 250 × 4 mm, 5 μm, 120 C18 AQ (Co. Maisch, Ammerbuch-Entringen, Germany). The mobile phase consisted of KH_2_PO_4_-Solution (6.8 g/l) with 2% acetonitrile, pH value adjusted to 2.4 with 20% *o*-phosphoric acid. The detection was carried out using a diode array detector at 220 nm with a reference wavelength of 500 nm.

### Data evaluation and statistical analysis

The relative levels of myoglobin redox states were calculated using the measured reflectance spectra, based on the studies of Krell et al. (2025, 2026). The statistical analyses, including calculation of mean values and standard errors, were calculated from the raw data using SPSS Statistics Version 31.0 (Co. IBM, Armonk, USA). First, the Shapiro-Wilk test was used to check all datasets for normal distribution (*p* > 0.05 = normal distribution confirmed). Since 19 out of the 30 datasets were not normally distributed, the statistical analysis was continued with non-parametrical tests. Tests for two independent groups (control and discolored) were chosen. To make comparisons between the groups the Mann-Whitney-*U* test and the Kruskal-Wallis tests were performed (*p* < 0.05 = significant difference between the two groups). Both tests showed the same results for the differences in all groups.

The correlation analysis was also calculated using SPSS Statistics Version 31.0. A nonparametric correlation analysis using Spearman’s rho (r_sp_) (*p* < 0.05 = significant correlation of two parameters) was performed.

The principal component analysis was calculated using Origin Pro 2023 (Co. OriginLab Corporation, Northhampton, MA, USA), using the principal component analysis app.

## Results and discussion

### Photographs of the samples

Photographs of the discolored samples and their respective controls before chemical analyses can be seen in Table 1.

**Table 1:**
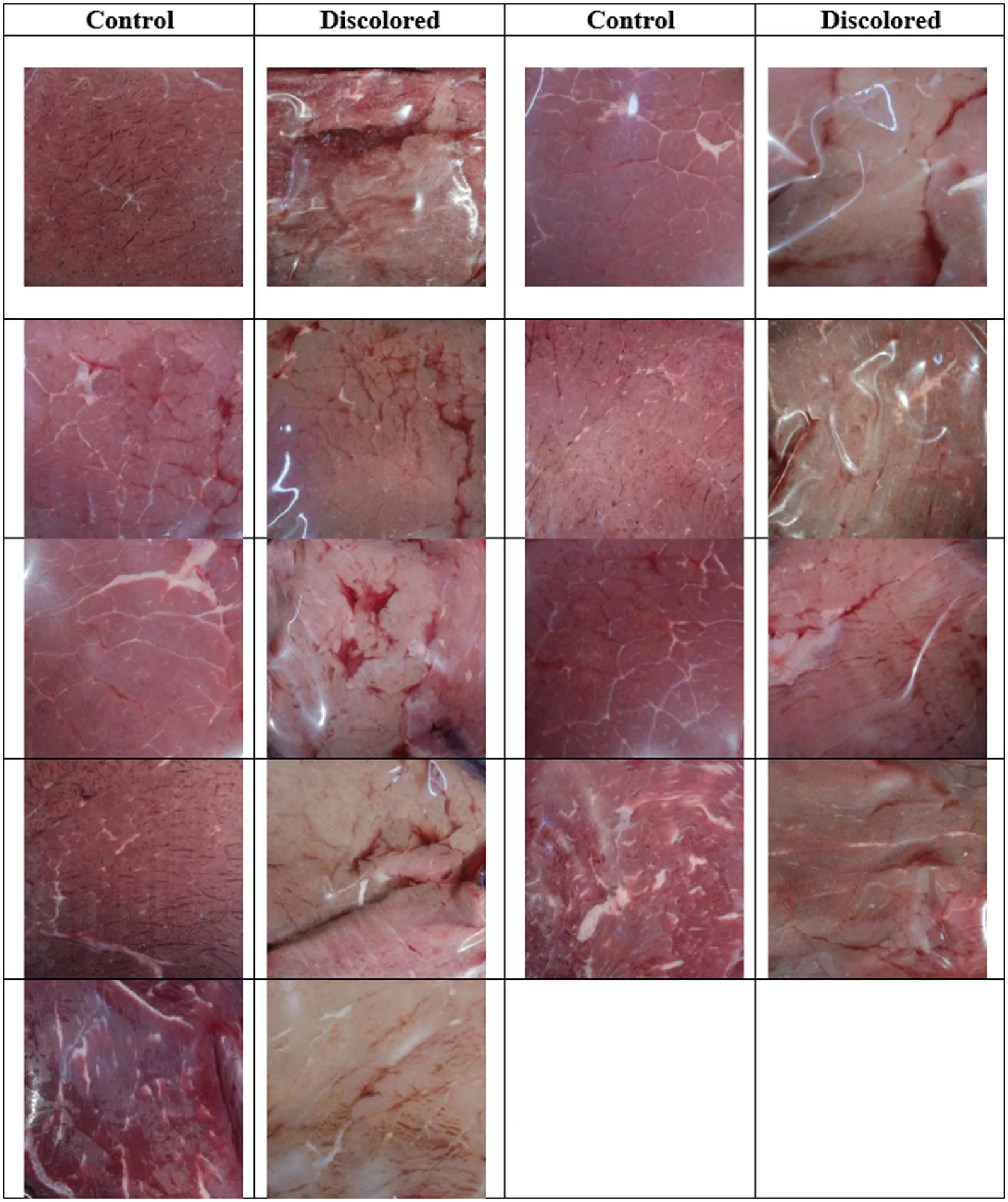
Photographs of the control group and discolored meat samples

The control samples appeared in dark and pale shades of violet and red. According to literature, this is typical for vacuum-packaged meat, as the dominant redox state of Mb should be DMb (King 2023; Krell et al. 2024; Mancini 2013). The discolored samples showed a very different color profile. They appeared lighter than the control samples and showed grey or brown discolorations that in some photos were uniformly distributed or concentrated in specific areas of the meat samples. The discolorations showed all the described characteristics in literature for MMb formation. Normally, irreversible discoloration should not occur in vacuum packages after a short packaging time of a few days (King 2023; Krell et al. 2024; Mancini 2013).

### Intrinsic parameters

The measured intrinsic parameters of the discolored and control samples are listed in Table 2. All given concentrations are expressed as mass of the analyzed substance/mass of the sample.

**Table 2:** Intrinsic parameters (relative oxy-, deoxy-, and metmyoglobin levels, color (L*, a*, b*), pH value, redox potential, total reducing ability, and the concentration of NAD_+_, NADH, *β*-carotene, and *α*-tocopherol) of the control group and discolored beef samples expressed as mean values *±* standard error

	Control	Discolored
L* (-)	38.63 ± 0.60^A^	43.21 ± 0.76^B^
a* (-)	11.21 ± 0.20^B^	9.42 ± 0.33^A^
b* (-)	10.39 ± 0.29^A^	12.48 ± 0.36^B^
Deoxymyoglobin (-)	1.30 ± 0.02^B^	1.13 ± 0.02^A^
Oxymyoglobin (-)	2.22 ± 0.03^B^	2.02 ± 0.04^A^
Metmyoglobin (-)	0.79 ± 0.01^A^	1.01 ± 0.03^B^
pH (-)	5.60 ± 0.03^A^	5.61 ± 0.02^A^
Lactate (mg/g)	3.90 ± 0.54^A^	5.60 ± 0.17^B^
Total Reducing Ability (-)	4.07 ± 0.03^A^	4.12 ± 0.03^A^
Redox Potential (mV)	457.00 ± 2.17^A^	459.87 ± 2.79^A^
NAD_+_ (µg/g)	0.14 ± 0.04^A^	0.30 ± 0.03^A^
NADH (µg/g)	5.41 ± 1.91^A^	4.03 ± 1.07^A^
*α*-Tocopherol (µg/g)	2.00 ± 0.33^A^	2.03 ± 0.18^A^
*β*-Carotene (µg/g)	0.68 ± 0.05^A^	0.64 ± 0.08^A^

Letters indicate significant differences (*p* < 0.05). Concentrations are expressed as mass of substance/mass of sample

As the photographs of the meat samples showed visible color deviations, logically these differences were also measured in the color analysis. There were significant (*p* < 0.05) differences between the discolored samples and the control group in each of the color values (L*a*b*). The color values of the control samples were 38.63 (L*), 11.21 (a*), and 10.39 (b*). The color values of the discolored samples were 43.21 (L*), 9.42 (a*), and 12.48 (b*). Thus, the measured color values characterized the discolored samples as lighter, less red and more yellow compared to the control (Wieser 2010; King 2023). As a measure to emphasize the discoloration of the discolored samples compared to the control: the Redness-index (a*/b*) is 1.08 for the control and 0.75 for discolored beef (Bu et al. 2022).

The color deviations of the discolored samples and the control group were also confirmed by the analysis of the relative level of myoglobin redox states. There were significant (*p* < 0.05) differences between the discolored samples and the control group in each of the Mb redox states DMb, OMb, and MMb. The control samples showed Mb levels of 1.30 (DMb), 2.22 (OMb), and 0.79 (MMb). The discolored samples showed values of 1.13 (DMb), 2.02 (OMb), and 1.01 (MMb). The DMb and OMb levels of the discolored samples were significantly (*p* < 0.05) lower than in the control group, while the MMb levels were significantly (*p* < 0.05) higher. These differences are logical, as the redox states of Mb convert into each other depending on the conditions to which the Mb state of meat is exposed. Therefore, a higher MMb level can only be possible if the OMb and/or DMb levels decrease (King 2023; Krell et al. 2025; Mancini 2013). The decreased redness compared to the control group fits to the decreased levels of DMb and OMb in the discolored samples, and the increased lightness and yellowness to the increased MMb levels. The behavior of the Mb redox states observed in this study, and the connection to the color values have been described in studies of Krell et al. (2024, 2025, 2026).

The only intrinsic factor that is not directly related to color, that showed significant differences (*p* < 0.05) between the control group and the discolored samples was the lactate concentration. The control group reached 3.90 mg/g (43.8 mmol/kg), while the discolored samples reached 5.61 mg/g (63.0 mmol/kg). Depending on the literature, these values are either lower or higher than the references, which, after at least 24 h postmortem report a wide range of lactate concentrations from 3.76 mg/g (42.2 mmol/kg) up to 8.9 mg/g (100 mmol/kg) (Devine and Dikeman 2014; Bendall 1978; Jerez-Timaure et al. 2022; Onopiuk et al. 2016). Therefore, abnormal lactate concentrations cannot be confirmed.

Although the influence of lactic acid bacteria on the differences in lactate concentrations of the samples cannot be totally excluded, the findings of a study of Poveda-Arteaga et al. under the same sampling conditions in the same slaughterhouse, did not observe a significant increase of lactic acid bacteria on the discolored samples (Poveda et al. 2025).

The other intrinsic factors, which are not directly related to color, showed no significant (*p* > 0.05) differences between the control group and the discolored samples. The pH value of the control group was 5.60, compared to 5.61 for the discolored samples. The pH values are in the normal range of ultimate pH (5.5–5.6) for beef according to literature (Devine and Dikeman 2014).

The difference in lactate concentration between the control and discolored samples, despite almost identical pH values, might be explained by the buffering capacity of meat (Devine and Dikeman 2014). Components such as amino acid and peptide side chains, as well as phosphates, bind H^+^ ions from lactic acid, forming lactate anions. This results in stable pH values as long as the buffer capacity is not exceeded (Devine and Dikeman 2014).

Assuming that the difference in lactate concentrations was not caused by microorganisms, the difference could hypothetically be attributed to factors that influenced the post-mortem glycolysis rate of the meat samples to different extents. One factor could be differences in the chilling rate of the samples (Bai et al. 2023; Kuffi et al. 2018; Lancaster et al. 2020). In the slaughterhouse, for chilling, carcasses are halved and hung in a cooling chamber. Therefore, muscles that are located deeper within the carcass maintain a higher temperature for a longer period of time. This allows for an increased glycolysis rate, which ultimately leads to higher lactate levels in these parts of the muscle (Bai et al. 2023; Kuffi et al. 2018; Lancaster et al. 2020; Liu et al. 2025). Another possible factor is an uneven distribution of mitochondria throughout the muscles (King et al. 2025; Ramos et al. 2021; Yen 2024). Muscle parts with increased mitochondrial concentrations exhibit increased glycolysis rates, which results in higher lactate concentrations in these areas (Liu et al. 2025; King et al. 2025; Ramos et al. 2021; Yen 2024). However, these possible causes of the differences in lactate concentration are hypothetical and not supported by the data from this study.

In literature, exogenous lactate is described as a color stabilizing substance in meat, since it can enhance the metmyoglobin reducing activity and glycolytic activity of the muscle tissue (Liu et al. 2025; Biswas and Mandal 2019; Mohan et al. 2010; Wang et al. 2022). Therefore, it seems unlikely that an increased lactate concentration itself is the reason for the discoloration.

There were no significant differences in the TRA or redox potential between the control group and the discolored samples (*p* > 0.05). The TRA and redox potential of the control group were 4.07 and 457.00 mV, compared to 4.12 and 459.87 mV for the discolored samples. In literature, TRAs of 0.40 to 0.80 and redox potentials between 260 and 530 mV (although with a different setups) are reported (Cucci et al. 2020; Ke et al. 2017; Sammel et al. 2002; Sepe et al. 2005). The measured TRAs do not confirm differences in the reducing systems of the samples. Also, different extents of oxidation in the samples cannot be confirmed according to the redox potential measured.

The discolored and control samples also showed no significant (*p* > 0.05) differences in the NAD^+^ and NADH concentrations. For the control group, the concentrations were 0.14 µg/g (NAD^+^) and 5.41 µg/g (NADH), respectively. For the discolored samples, the concentrations were 0.3 µg/g (NAD^+^) and 4.03 µg/g (NADH). A comparison with literature revealed concentration ranges of 5.97 µg/g to 126 µg/g (NAD^+^) and 7.99 µg/g to 19.29 µg/g (NADH) depending on the storage time (Mitacek et al. 2019; Madhavi and Carpenter 1993). The measured NAD^+^ and NADH values of both groups are lower than those, which might be connected to the deviating measurement methods. Hypothetically, another reason for the lower values is the treatment of the samples before measurement. Because the samples were frozen at − 18 °C, transported, and then thawed for 2 days at 2 °C, NAD^+^ and NADH degradation might have occurred due to cell membrane damage, the connected drip loss, and oxidation processes (Ben Abdallah et al. 1999; Bouchendhomme et al. 2022). The thawing time of 2 days before analysis may have led to the degradation of metabolites and endogenous enzymes (Bouchendhomme et al. 2022; Kwon et al. 2022). Although the measured NAD_+_/NADH values seem to be different, the standard deviations were high which resulted in insignificant (*p* > 0.05) differences. Due to the lack of differences in the NAD_+_ and NADH concentrations, differences in the enzymatic reducing capacity or the extent of completed reducing processes cannot be confirmed.

The investigated antioxidants *α*-tocopherol and *β*-carotene also showed no significant (*p* > 0.05) concentration differences between the discolored and control samples. The concentrations of the control group were 2.00 µg/g (*α*-tocopherol) and 0.68 µg/g (*β*-carotene). The concentrations of the discolored group were 2.03 µg/g (*α*-tocopherol) and 0.64 µg/g (*β*-carotene). In literature, concentrations of *α*-tocopherol (1.90 to 6.00 µg/g) and *β*-carotene (0.02 to 1.73 µg/g) have been reported (Nassu et al. 2011; Jin et al. 2015; Terevinto et al. 2023; Muramoto et al. 2003; Li et al. 2012). Therefore, both measured parameters were in the expected range. Similar antioxidant concentrations indicate similar oxidation processes, since excessive oxidation would decrease antioxidant content as well (Terevinto et al. 2023).

The analysis of the intrinsic parameters showed clear differences in the color and myoglobin redox states, but none of the investigated parameters could be determined as the cause of the discolorations. Hypothetical possibilities are that the causing parameter was simply not analyzed, that the discolorations are caused by multiple parameters that amplify each other, or that the discoloration is not caused by intrinsic factors but extrinsic factors, such as oxygen pockets formed in the packaging by faulty evacuation in the vacuum packages (Krell et al. 2024, 2025, 2026).

The limitations of this study include the unforeseeable and scarce occurrence of the discolored samples. Even with a sampling period of four months, only a few discolored packages were identified in the slaughterhouse. A larger sample size would be beneficial. Due to the processes in the slaughterhouse, it was not possible to backtrack the individual meat pieces in one package. Therefore, it could not be assured that the control samples were from the same animal. It could only be assured that the samples were from the same group of animals, meaning that their raising conditions, breed, and sex were identical.

### Correlation analysis

Due to the limited significance obtained from the Mann-Whitney-*U* and Kruskal-Wallis tests, a correlation analysis was used to examine the relationships between the parameters more closely and improve the understanding of their influence. The results of the correlation analysis can be seen in Table 3. Significant correlations (*p* < 0.05) between two variables are marked with *. Spearman-Rho correlation coefficients r_sp_ > 0 describe a positive correlation of the parameters, while r_sp_ < 0 describe a negative correlation (Schober et al. 2018). The effect strength of the correlation can be described by the value of r_sp_. According to Schober and colleagues (Schober et al. 2018), the effects can be distinguished in neglectable (r_sp_ < 0.1), weak (r_sp_ = 0.10–0.39), moderate (r_sp_ = 0.40–0.69), strong (r_sp_ = 0.70–0.89), and very strong (r_sp_ > 0.90). It should be noted that the sharp distinction between the effect sizes should be used carefully, and in consideration of the dataset, as the distribution of the data can also influence the outcome. To understand the distribution of the correlation data the confidence intervals of the spearman coefficients are listed in the Appendix (Table 4).

**Table 3:**
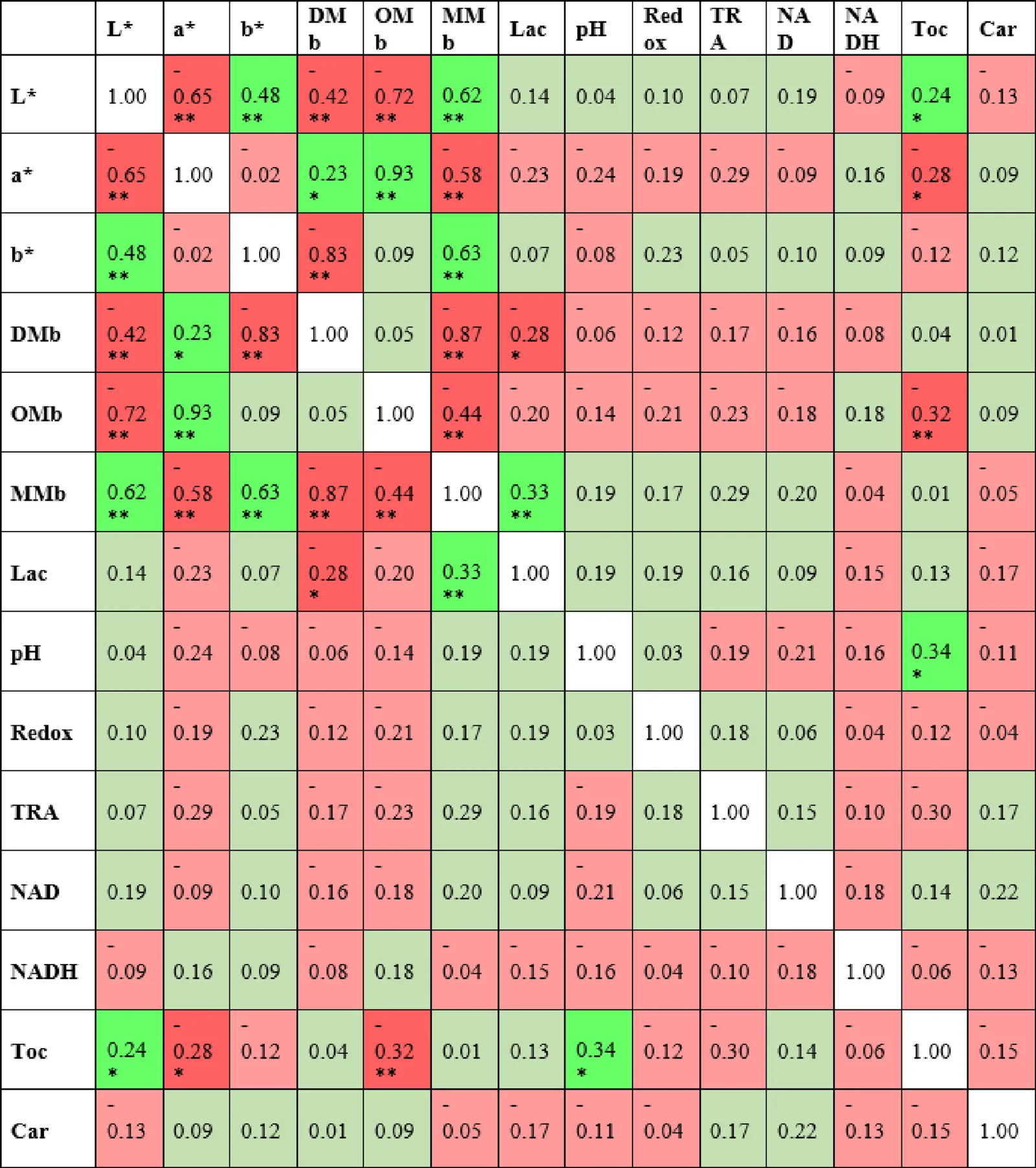
Spearman-Rho correlation coefficients *r*_*sp*_ of the measured parameters

Positive values (pale green) indicate positive correlations, negative values (pale red) negative correlations between two variables. Values marked with *(*p* < 0.05) or **(*p* < 0.01) show significant correlations between two variables and are emphasized in bright green or red

The significant (*p* < 0.05) correlations can be divided into two groups: one group describing the correlation of color and myoglobin with themselves, and the other group describing color/myoglobin correlation to other intrinsic parameters

The positive correlations of the first group were L*–b* (r_sp_ = 0.48, moderate), L*–MMb (r_sp_ = 0.62, moderate), a*–DMb (r_sp_ = 0.23, weak), a*–OMb (r_sp_ = 0.93, very strong), and b*–MMb (r_sp_ = 0.63, moderate). Thus, the L* and b* values increased with the MMb levels and were associated with discoloration. In contrast, the a* value correlated partly with DMb and mostly OMb levels and was associated with normal color.

The negative correlations of the first group were L*–a* (r_sp_ = − 0.65, moderate), L*–DMb (r_sp_ = − 0.42, moderate), L*–OMb (r_sp_ = − 0.72, strong), a*–MMb (r_sp_ = − 0.58, moderate), b*–DMb (r_sp_ = − 0.83, strong), DMb–MMb (r_sp_ = − 0.87, strong), and OMb–MMb (r_sp_ = − 0.44, moderate). These correlations presented the inverse picture compared to the positive correlations as the parameters associated with discoloration, L*, b* and MMb negatively correlated with parameters that were associated with normal color, a*, DMb, and OMb.

The observations of the parameter interactions of the first group align with studies of Krell et al. (2024, 2025).

While the correlations in the first group give the information which color values and myoglobin redox states are the direct reason for the discoloration, the correlations in the second group may give information about the underlying reasons for the discoloration caused by deviations in the intrinsic factors not directly related to color.

The significantly positive correlations in the second group were L* and *α*-tocopherol (r_sp_ = 0.24, weak), MMb–lactate (r_sp_ = 0.33, weak), and pH–*α*-tocopherol (r_sp_ = 0.34, weak). The correlations including the pH value and *α*-tocopherol should be examined carefully since there were insignificant differences between the groups for both parameters. However, the correlation between MMb and lactate is more important, since both parameters undergo significant changes. Though the effect of the correlation is weak, there was an association between discoloration and increased lactate concentration to some extent. This correlation cannot be confirmed as a causation, as the literature reports that lactate is a meat color stabilizing substance rather than a discoloration-promoting one. The color stabilizing effect of lactate has been described as an increased lactate dehydrogenase activity, which leads to increased NADH formation and therefore higher metmyoglobin reducing activity (Biswas and Mandal 2019; Wang et al. 2022).

The negative correlations in the second group were a*–*α*-tocopherol (r_sp_ = − 0.28, weak), DMb–lactate (r_sp_ = − 0.28, weak), and OMb–*α*-tocopherol (r_sp_ = − 0.32, weak). Again, the correlations including *α*-tocopherol should be considered carefully, since there were insignificant differences between the groups. The association of lactate with discoloration is also shown by the negative correlation to DMb, because it is connected to the normal color of meat. Again this connection cannot be considered as a proof of cause for the discolorations, as lactate is a meat color stabilizer (Biswas and Mandal 2019; Wang et al. 2022).

The correlation analysis clearly identified the color and Mb redox states that are associated with discoloration and provided a statistical context, which was not possible with a simple statistical comparison of the two groups. However, the correlation analysis has limitations. Parameters showing very small differences between the groups require careful interpretation (Schober et al. 2018).

### Principal component analysis

**Fig. 1:**
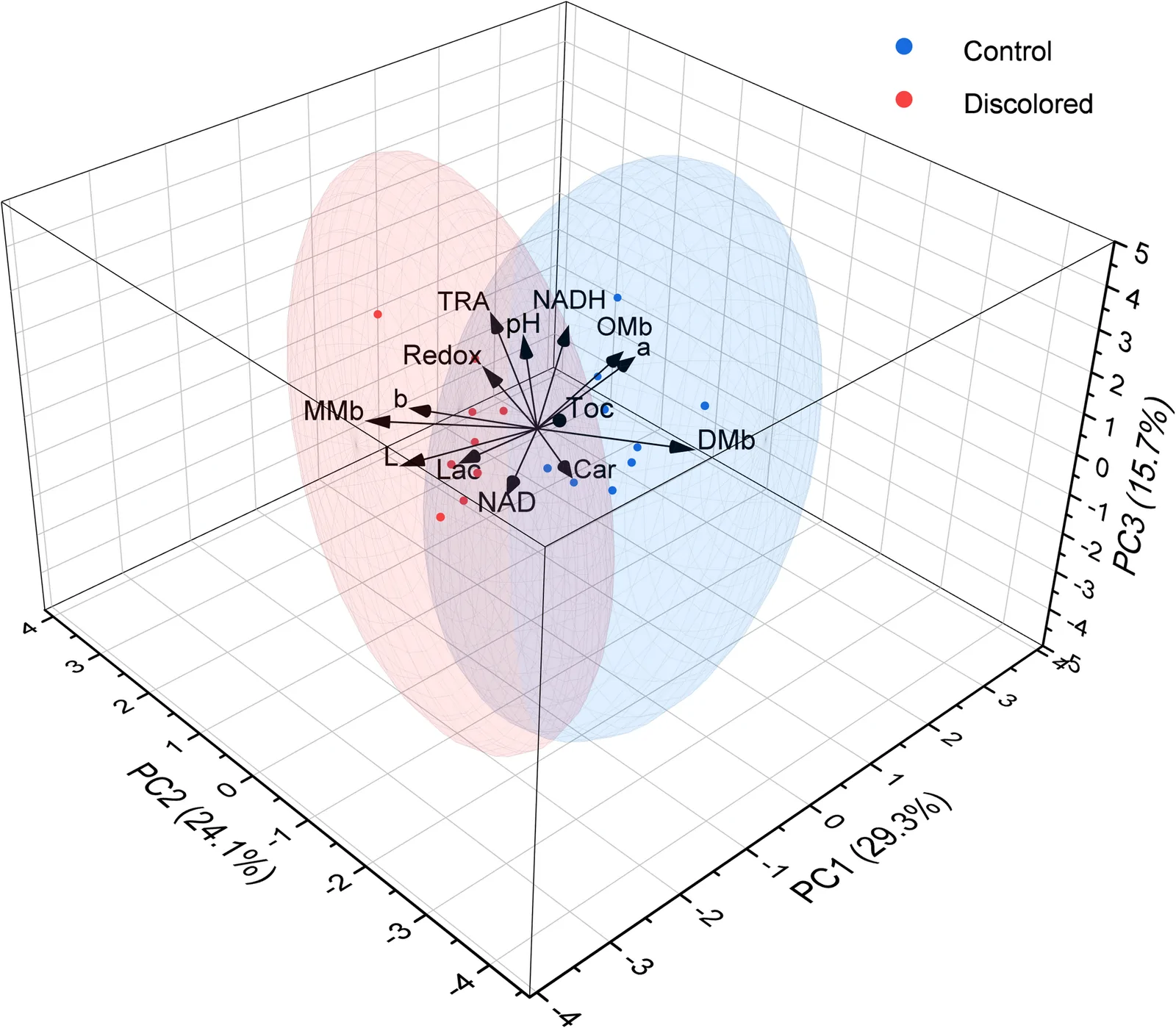
Principal component analysis of the control and discolored beef samples. The three principal components are PC1, PC2 and PC3. The correlation matrix includes the oxy- (OMb), deoxy- (DMb), and metmyoglobin (MMb) levels, color (L*, a*, b*), pH value, redox potential (redox), total reducing ability (TRA), and the concentration of NAD_+_, NADH, *β*-carotene, and *α*-tocopherol

To get an even deeper understanding of the investigated parameters and their role in the discoloration process, the principal component analysis (PCA) was used. The PCA can be seen in Fig. 1. In the PCA, each point represents one of the nine control or discolored samples and is placed in the 3D coordination system according to the mean values of all the respective sample’s measured parameters. The arrows are the so-called loadings, which are vectors that indicate the correlation between parameters. The PCA displays parameter correlations in the context of the data points. This enables a visual understanding of the differences between the two groups, while also accounting for the effects of the parameters (Greenacre et al. 2022). The factor loadings of the PCA can be seen in Table 5 in the Appendix.

The total cumulative variance of 69.1% consists of PC1 (29.3%), PC2 (24.1%), and PC3 (15.7%). Thus, the PCA can explain 69.1% of the variance with 3 components, while 30.9% remains random or unexplained (Greenacre et al. 2022). A comparison with the literature shows that this PCA results in a relatively precise description of the data due to its higher or similar cumulative variance (Greenacre et al. 2022; Schumacher et al. 2025; Mwove et al. 2018; Boligon et al. 2016).

The PCA allows for a clear distinction between the control and discolored samples, as seen in the two separate clusters of data points. The vectors of the parameters that are associated with each group point in the direction of its respective data point clusters. According to the PCA, the parameters TRA, redox potential, L* and b* value, MMb and lactate, and NAD_+_ were associated with discoloration. The parameters OMb, DMb, a* value, *β*-carotene, *α*-tocopherol, and NADH were associated with normal color. The pH value was not associated with either group. For redox potential, L*, a*, b*, DMb, OMb, MMb, NAD_+_/NADH, *β*-carotene and *α*-tocopherol, these findings align with the literature. However, literature does not confirm the connection between increased TRAs or lactate content and discoloration (Krell et al. 2024; Cucci et al. 2020; Tuell et al. 2021; Mitacek et al. 2019; Jin et al. 2015; Mohan et al. 2010; Madhavi and Carpenter 1993; Nassu et al. 2011). Similarly, to the correlation analysis, the very small insignificant changes of some parameters might have led to a statistical overestimation of the effects which could explain why TRA was associated with discoloration.

Although the simple statistical comparison using the Mann-Whitney-*U* and Kruskal-Wallis tests showed significant differences in all the analyzed parameters, the PCA clearly differentiated between the control and discolored samples. However, due to the limited sample size and therefore lack of statistical significance, these results should be considered carefully. The associations of the analyzed parameters mostly aligned with the literature. Since the sample size and independent sampling were important limiting factors of this study, it would be highly beneficial to collect more data in future studies with an increased sample size and, if possible, a paired sampling approach. With a sufficient sample size, developing a multivariate indicator model for beef discolorations might be possible.

## Conclusion

In the photos, and all three used statistical methods (Mann-Whitney-*U*/Kruskal-Wallis test, correlation analysis and PCA) the differences in color and the connected changes in the myoglobin redox states between the control and discolored beef samples are observable, measurable and align with the literature. However, the analysis of intrinsic parameters not directly connected to the color revealed different results, depending on the statistical method used. Mann-Whitney-*U*/Kruskal-Wallis test only revealed significantly higher lactate concentrations in the discolored samples. The only logical correlations obtained in the spearman correlation analysis showed increased lactate concentrations as the only affecting parameter of the discoloration. The PCA was able to generate a more complete picture, but the statistical limitations of the dataset need to be considered. Increased values of TRA, redox potential, lactate and NAD^+^ were associated with the discolored samples. The PCA was able to differentiate the control and discolored sample groups and put the influence of the analyzed parameters into context.

The hypothesis of this study, that one or more of the intrinsic factors in the discolored muscle tissue deviated from the control muscle tissue and that one or more of these factors caused the discoloration, can only be partially confirmed. The lactate concentration significantly deviated in the discolored samples, but a conclusive causation of the discoloration exclusively due to increased lactate could not be confirmed. Even though the cause of the discoloration could not be identified by the investigated parameters, the data reported in this study and the interactions between the intrinsic parameters could be the foundation for further research to reveal the causes of the discoloration.

Future trials should expand the sample collection period to one year and/or investigate multiple slaughterhouses to increase the sample size and, consequently, the statistical significance of the data. Additionally, analyzing additional intrinsic and extrinsic parameters should be considered. The ultimate aim should be to develop an extensive, statistically highly significant, multivariate indicator model for beef discoloration that includes all possible parameters.

## Data Availability

The Data is available on request.

## Appendix

See Tables 4, 5 here.

**Table 4:** Spearman-Rho (r_sp_) correlation coefficients with significances and confidence intervals (CI)

Parameters	*r*_sp_	Significance	95% CI lower bound	95% CI upper bound
L*–a*	− 0.654	< 0.001	− 0.761	− 0.513
L*–b*	0.480	< 0.001	0.297	0.628
L*–DMb	− 0.421	< 0.001	− 0.582	− 0.229
L*–OMb	− 0.719	< 0.001	− 0.809	− 0.598
L*–MMb	0.620	< 0.001	0.469	0.736
L*–Lactate	0.136	0.256	− 0.106	0.362
L*–pH	0.040	0.775	− 0.238	0.312
L*–Redox	0.097	0.575	− 0.249	0.420
L*–TRA	0.070	0.686	− 0.274	0.398
L*–NAD_+_	0.189	0.113	− 0.052	0.408
L*–NADH	− 0.087	0.467	− 0.319	0.154
L*–*α-*Tocopherol	0.243	0.040	0.005	0.455
L*–*β*-Carotene	− 0.126	0.292	− 0.353	0.116
a*–b*	− 0.015	0.886	− 0.228	0.198
a*–DMb	0.226	0.032	0.013	0.419
a*–OMb	0.926	< 0.001	0.887	0.951
a*–MMb	− 0.575	< 0.001	− 0.702	− 0.412
a*–Lactate	− 0.230	0.052	− 0.444	0.008
a*–pH	− 0.238	0.083	− 0.482	0.040
a*–Redox	− 0.188	0.272	− 0.494	0.160
a*–TRA	− 0.294	0.082	− 0.574	0.048
a*–NAD_+_	− 0.091	0.446	− 0.323	0.150
a*–NADH	0.159	0.181	− 0.082	0.383
a*–*α-*Tocopherol	− 0.281	0.017	− 0.486	− 0.046
a*–*β*-Carotene	0.092	0.443	− 0.150	0.323
b*–DMb	− 0.827	< 0.001	− 0.884	− 0.745
b*–OMb	0.094	0.380	− 0.122	0.301
b*–MMb	0.625	< 0.001	0.476	0.740
b*–Lactate	0.068	0.573	− 0.173	0.301
b*–pH	− 0.082	0.556	− 0.349	0.198
b*–Redox	0.231	0.175	− 0.116	0.527
b*–TRA	0.048	0.779	− 0.294	0.380
b*–NAD_+_	0.098	0.412	− 0.143	0.329
b*–NADH	0.092	0.440	− 0.149	0.324
b*–*α-*Tocopherol	− 0.118	0.324	− 0.346	0.124
b*–*β*-Carotene	0.119	0.320	− 0.123	0.347
DMb–OMb	0.046	0.668	− 0.169	0.256
DMb–MMb	− 0.871	< 0.001	− 0.914	− 0.807
DMb–Lactate	− 0.275	0.019	− 0.482	− 0.040
DMb–pH	− 0.059	0.673	− 0.329	0.220
DMb–Redox	− 0.121	0.482	− 0.441	0.226
DMb–TRA	− 0.171	0.320	− 0.480	0.177
DMb–NAD_+_	− 0.164	0.169	− 0.387	0.077
DMb–NADH	− 0.078	0.515	− 0.310	0.163
DMb–*α-*Tocopherol	0.036	0.767	− 0.204	0.272
DMb–*β*-Carotene	0.005	0.964	− 0.233	0.243
OMb–MMb	− 0.439	< 0.001	− 0.597	− 0.250
OMb–Lactate	− 0.195	0.101	− 0.414	0.045
OMb–pH	− 0.138	0.319	− 0.398	0.142
OMb–Redox	− 0.208	0.222	− 0.510	0.139
OMb–TRA	− 0.228	0.180	− 0.525	0.118
OMb–NAD_+_	− 0.181	0.127	− 0.402	0.060
OMb–NADH	0.184	0.121	− 0.056	0.405
OMb–*α-*Tocopherol	− 0.319	0.006	− 0.518	− 0.087
OMb–*β*-Carotene	0.088	0.461	− 0.153	0.320
MMb–Lactate	0.332	0.004	0.102	0.529
MMb–pH	0.187	0.175	− 0.093	0.440
MMb–Redox	0.172	0.317	− 0.176	0.481
MMb–TRA	0.289	0.088	− 0.054	0.571
MMb–NAD_+_	0.199	0.094	− 0.041	0.417
MMb–NADH	− 0.037	0.759	− 0.273	0.203
MMb–*α-*Tocopherol	0.008	0.945	− 0.230	0.246
MMb–*β*-Carotene	− 0.052	0.665	− 0.287	0.189
Lactate–pH	0.186	0.178	− 0.094	0.439
Lactate–Redox	0.190	0.267	− 0.157	0.496
Lactate–TRA	0.162	0.346	− 0.186	0.473
Lactate–NAD_+_	0.093	0.435	− 0.148	0.324
Lactate–NADH	− 0.149	0.212	− 0.374	0.093
Lactate–*α-*Tocopherol	0.131	0.274	− 0.111	0.358
Lactate–*β*-Carotene	− 0.169	0.156	− 0.392	0.072
pH–Redox	0.032	0.852	− 0.309	0.366
pH–TRA	− 0.194	0.256	− 0.499	0.153
pH–NAD_+_	− 0.214	0.120	− 0.462	0.065
pH–NADH	− 0.163	0.238	− 0.420	0.117
pH–*α-*Tocopherol	0.336	0.013	0.067	0.559
pH–*β*-Carotene	− 0.105	0.451	− 0.369	0.176
Redox–TRA	0.182	0.287	− 0.165	0.490
Redox–NAD_+_	0.058	0.736	− 0.285	0.388
Redox–NADH	− 0.037	0.832	− 0.370	0.305
Redox–*α-*Tocopherol	− 0.123	0.473	− 0.442	0.223
Redox–*β*-Carotene	− 0.035	0.841	− 0.368	0.306
TRA–NAD_+_	0.147	0.391	− 0.200	0.462
TRA–NADH	− 0.094	0.585	− 0.418	0.251
TRA–*α-*Tocopherol	− 0.299	0.077	− 0.578	0.043
TRA–*β*-Carotene	0.174	0.310	− 0.174	0.483
NAD_+_–NADH	− 0.178	0.134	− 0.400	0.062
NAD_+_–*α-*Tocopherol	0.144	0.227	− 0.097	0.370
NAD_+_–*β*-Carotene	0.215	0.070	− 0.025	0.431
NADH–*α-*Tocopherol	− 0.063	0.601	− 0.297	0.178
NADH–*β*-Carotene	− 0.125	0.297	− 0.352	0.117
*α-*Tocopherol–*β*-Carotene	− 0.149	0.212	− 0.374	0.093

**Table 5:** Principal component analysis factor loadings and percentage of variance explained for the intrinsic factors of the control and discolored beef samples

Variables	Factor 1	Factor 2	Factor 3
Lactate	− 0.311	− 0.039	0.084
pH	− 0.024	0.045	0.559
Redox Potential	− 0.087	0.147	0.342
Total Reducing Ability	0.147	0.408	0.294
Deoxymyoglobin	0.181	− 0.473	0.106
Metmyoglobin	− 0.351	0.341	0.065
Oxymyoglobin	0.437	0.129	0.033
*L**	− 0.346	0.187	− 0.093
*a**	0.451	0.091	0.016
*b**	− 0.092	0.487	− 0.188
NADH	0.300	0.227	0.231
NAD^+^	− 0.256	− 0.185	− 0.038
*α*-Tocopherol	− 0.150	− 0.294	0.427
*β*-Carotene	0.133	− 0.011	− 0.423
Variance explained	29.3	24.1	15.7
